# Impact of donor site on fat graft survival in autologous fat transfer to the breast: A systematic review

**DOI:** 10.1016/j.jpra.2026.06.002

**Published:** 2026-06-17

**Authors:** Kyrah S. Goeree, Wessel B.W. van der Venne, Edith Visser, Andrzej A. Piatkowski, Chantal M. Mouës-Vink

**Affiliations:** a Department of Plastic, Reconstructive and Hand surgery, Frisius Medical Centre Leeuwarden, Henri Dunantweg 2, 8934 AD Leeuwarden, The Netherlands; b NUTRIM School for Nutrition, and Translational Research in Metabolism, Maastricht University, P.O. Box 616, Universiteitssingel 40, 6229 ER Maastricht, The Netherlands; c Department of Plastic, reconstructive, and hand surgery, Maastricht University Medical Center+, P.O. box 5800, 6202 AZ Maastricht, The Netherlands; d Department of Epidemiology, Frisius Medical Centre Leeuwarden, Henri Dunantweg 2, 8934 AD Leeuwarden, The Netherlands

**Keywords:** Breast reconstruction, Fat transfer, Lipofilling, Graft retention, Donor site

## Abstract

**Background:**

Autologous fat transfer has gained recognition as total breast reconstruction. Application remains constrained by unpredictable fat graft survival. Donor site selection may influence fat survival, but evidence remains inconsistent and consensus is lacking. This systematic review evaluated the impact of donor site selection on fat graft survival following autologous fat transfer to the breast.

**Methods:**

MEDLINE, Cochrane Library, Embase, and Scopus were searched up to October 27, 2025. Eligible studies included randomized controlled trials and cohort studies reporting donor site and graft survival. Risk of bias was assessed using ROBINS-I V2. Meta-analyses were conducted using a random-effects model.

**Results:**

Seven studies comprising 291 patients were included. Fat was harvested from the abdomen (n=211) or thighs (n=80). No study specifically addressed total breast reconstruction. Pooled mean graft survival was 56.04% (95% CI, 41.57-70.51; *I^2^* = 99.8%) at 12 months postoperatively for the abdomen and 34.13% (95% CI, 30.51-37.76; *I^2^* = 15.2%) at 6 months postoperatively for the thighs. All studies had a serious or critical risk of bias.

**Discussion:**

Although fat graft survival in autologous fat transfer to the breast appears higher when fat is harvested from the abdomen compared to the thighs, these findings should be interpreted with caution. Due to low-quality evidence, time bias and substantial study heterogeneity, no donor site can be recommended based on fat graft survival alone. High-quality, standardized research is needed to clarify the role of donor site in graft survival as such evidence could guide surgical decision-making and improve outcomes in total breast reconstruction.

## Introduction

The use of autologous fat transfer (AFT) to the breast has expanded over recent decades. Initially applied by plastic surgeons to correct contour deformities,^1^^,^^2^ autologous fat transfer is now increasingly used to reconstruct larger defects. A Dutch multicenter randomized controlled trial (RCT) demonstrated the safety and efficacy of total breast reconstruction after breast cancer using AFT, leading to its inclusion in the Dutch basic health insurance in 2023.^3^^,^^4^

A total breast reconstruction using AFT can result in breasts with a natural shape, satisfactory sensibility and natural behavior, such as ptosis and responsiveness to weight fluctuations.^5^, ^6^, ^7^ The procedure is minimally invasive and associated with a low risk of procedure-related complications.^4^^,^^5^^,^^8^ Unlike flap-based breast reconstruction, AFT does not require large incisions resulting in extensive scarring or the availability of a single dedicated donor site. Thereby improving donor-site satisfaction and expanding its suitability across a wider patient population.^9^^,^^10^ However, multiple surgeries are required to reach the desired breast volume, resulting in a long and intensive treatment trajectory.^11^

Numerous attempts have been made to improve volumetric results of AFT. A variation of harvesting, processing and reinjection techniques have been proposed. However, no consensus exists regarding the superior techniques and thus a considerable variation persists in clinical practice.^12^^,^^13^ Enriching the lipoaspirate, for example with stromal vascular fraction (SVF) or platelet-rich plasma (PRP), might improve fat graft survival.^14^ However, this is not common practice and therefore there remains a lack of standardized protocols.^15^ Aside from technique, graft survival is influenced by the volume of transplanted fat: larger volumes are associated with greater retention.^16^ Yet, excessive volume is associated with harmful interstitial pressure and reduced graft survival.^17^

The unpredictability of the volumetric outcomes consequently remains a limitation of AFT, with reported fat graft survival rates varying widely. Regarding partial breast reconstructions, a 2015 systematic review by Yu et al.^18^ found survival rates ranging from 15% to 83%. A 2018 meta-analysis by Krastev et al.^2^ reported a survival of 52.4% at one year, which was limited by high heterogeneity. More recently, Wederfoort et al.^11^ reported a 12-month graft survival rate of 37.1% following total breast reconstruction using AFT.

A potential determinant for fat graft survival is the donor site from which adipose tissue is harvested. Fat graft viability is partly dependent on adipose-derived stem cells (ADSC), which play a key role in promoting adipogenesis and angiogenesis.^17^^,^^19^ Padoin et al.^20^ demonstrated that ADSC-concentrations vary among anatomical donor sites, suggesting that donor site selection may influence graft survival. However, in the absence of consensus on an optimal donor site, selection is currently guided by availability of adipose tissue and patient preference.^13^

With the expanding use of AFT in breast reconstruction, optimizing graft survival and improving outcome predictability have become increasingly important. However, evidence regarding the influence of donor site selection on fat graft survival in breast AFT remains inconclusive. Therefore, this systematic review aims to evaluate the impact of the donor site selection on the fat graft survival following AFT to the breast.

## Methods

### Study design

This systematic review adhered to the Preferred Reporting Items of Systematic Reviews and Meta-Analyses (PRISMA) statement (see Supplemental Table 5).^21^

### Search strategy

A comprehensive literature search was conducted in MEDLINE, Cochrane Library, Embase, and Scopus to identify all eligible studies. The search strategy was developed in collaboration with a medical librarian, and the last search was conducted on October 27, 2025. Search terms are presented in Table 1, and the comprehensive search strategy is provided in Supplemental Table 1. No restrictions were applied for publication date, publication status and language. Publications of editorials, comments and letters were restricted from the search.

**Table 1:** Search terms MEDLINE.

(exp Adipose Tissue/tr or exp Adipocytes/tr or ((exp "Autografts"/ or exp "tissue and organ harvesting"/ or exp "Transplantation, Autologous"/) and (exp "Adipose Tissue"/ or exp "Adipocytes"/ or (fat or fats or lipo* or lipid* or adipose).ti,ab,kf.)) or exp "lipectomy"/ or (((fat or fats or lipo* or lipid* or adipose) adj3 (trans* or injection* or graft* or autograft* or autotrans*)) or lipoinjection* or lipomodel* or lipotrans* or lipoaspirat* or liposuct* or lipectom* or lipofil* or AFT).ti,ab,kf.) and (exp "Mammaplasty"/ or exp Breast/su or (((Reconstruct* or Augment* or Oncoplasti*) adj4 Breast*) or mammoplast* or mastectom* or mastopex*).ti,ab,kf. or (exp "Transplant Donor Site"/ or ("donor site*" or "harvest site*").ti,ab,kf.))

### Study selection

Duplicates were removed using Endnote Reference Management Tool version X9 and verified manually. Covidence was used for title and abstract screening, full-text assessment and data extraction. All original studies reporting on fat graft survival and donor site characteristics in women, aged 18 years or older, undergoing large-volume AFT to the breast were considered for inclusion. Two reviewers (KG and WV) independently performed eligibility assessment using predefined inclusion and exclusion criteria (Table 2). Discrepancies were resolved through discussion until consensus was reached.

**Table 2:** Eligibility criteria.

Inclusion criteria	Human subjects Age ≥ 18 years old Lipofilling of the breast, both reconstructive and cosmetic Donor sites reported Fat graft survival rate or fat graft resorption rate reported Average volume of lipofilling ≥ 100 ml

Exclusion criteria	Animal studies, in vitro studies Children No description of donor sites. No description of fat graft survival or fat graft resorption. Other recipient sites than the breast Research protocol Systematic reviews, meta-analysis, editorials, comments, letters No full text available Average volume of lipofilling < 100 ml

### Study outcome

Main study outcome was fat graft survival in the reconstructed or augmented breast using AFT in relation to the donor site. Fat graft survival was defined as the percentage of injected fat retained relative to total volume of fat injected.

### Data extraction

Two reviewers (KG and WV) independently extracted relevant data using a predefined data extraction form (Supplemental Table 2), and discrepancies were resolved through discussion until consensus was reached. Authors were contacted to ask for raw, anonymous data. However, authors either did not reply or were unable to distribute the data.

### Risk of bias

All studies were assessed using the ROBINS-I tool Version 2 (2024).^22^ In the search, no randomized studies comparing donor sites were identified. Therefore, groups from the studies were reviewed individually and also assessed for risk of bias individually. Extracted groups from RCTs were therefore assessed as non-randomized studies. Two reviewers (KG and WV) assessed the studies independently and were checked by a third reviewer (EV). Disagreements were resolved through discussion.

### Data analysis

Measurement units were standardized. Continues variables reported in median (range) values were translated to mean (standard deviation, SD) using the standard equations used for meta-analyses.^23^ Fat graft survival rates were calculated as the percentage of retained fat volume relative to total injected volume. Resorption rates (percentage) were translated to survival rates by subtracting the resorption rate from 100%.

Statistical analysis was performed using R version 4.5.2 (R Foundation for Statistical Computing, Vienna, Austria). Heterogeneity among studies was assessed using *I^2^* and the random effects model was assumed. For each donor site, pooled mean graft survival rate with 95% confidence intervals (95%-CI) were estimated and presented in a forest plot.

## Results

### Study selection

**Fig. 1:**
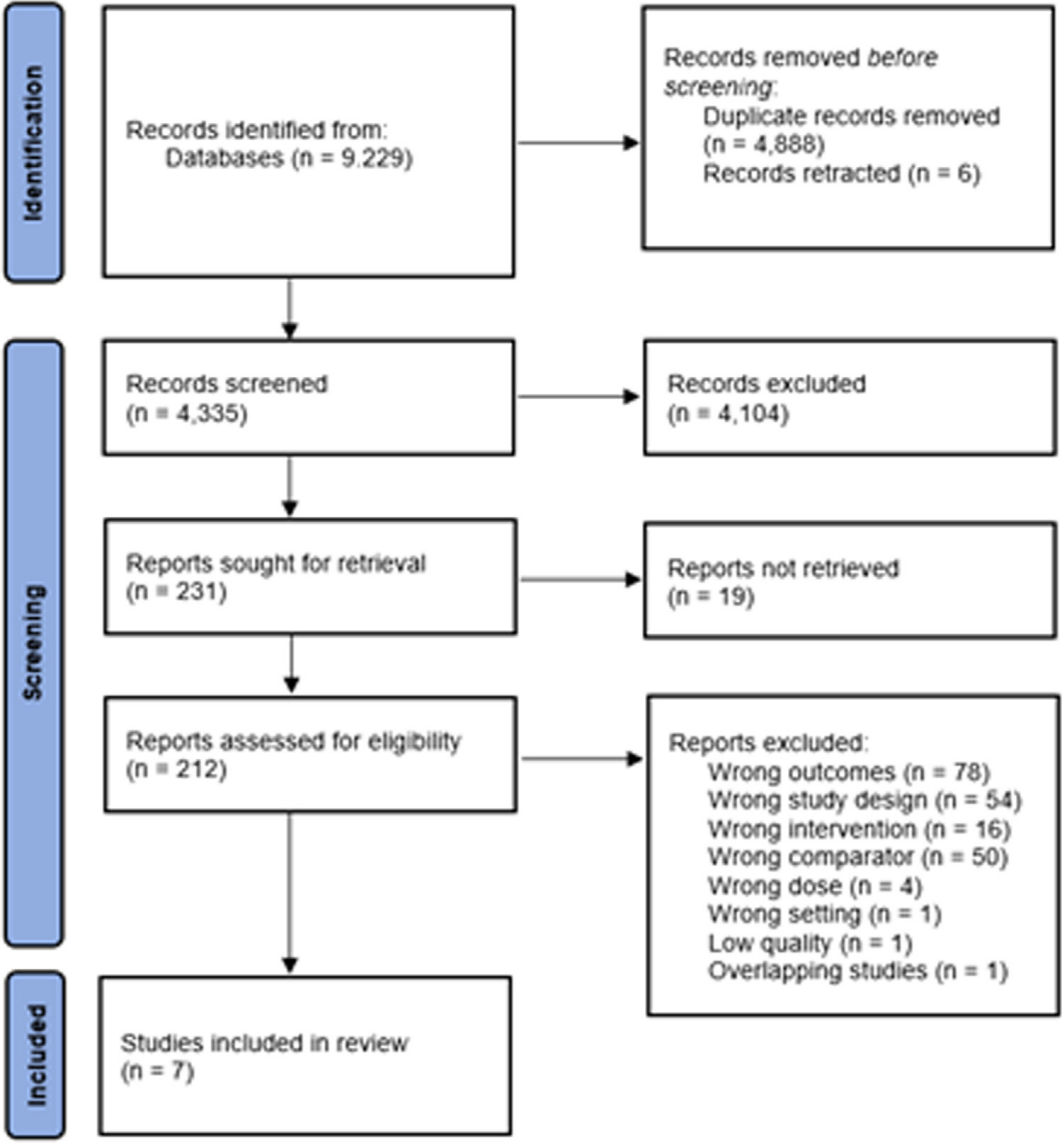
Study selection process.

Study selection process is summarized in Fig. 1. Database searching identified 9,229 records, of which 4,335 remained after removal of duplicates. Following title and abstract screening, 212 full-text articles were assessed for eligibility, resulting in nine eligible studies. Subsequently, one study was excluded due to the insufficient methodical quality and one due to possible overlap with another study by the same authors.^24^^,^^25^ Data from the remaining seven studies were extracted and summarized in Table 3 (see also Supplemental Table 3 and 4).

**Table 3:** Summary of extracted data from the included studies.

	Study design				Study population				Intervention							Outcome		
Donor site used	First author, publication year	Study design	Country	Start year – end year	Number of included patients	Age, years (mean, SD)	BMI, kg/m^2^ (mean, SD)	Radiotherapy included?	Surgery type	Enrichment	Indication (reconstructive, augmentation)	Harvesting technique (manuel/device)	Processing technique	Number of sessions (mean)	Total injection volume, ml (mean, SD)	Measurement of outcome	Time of measurement, months	Fat graft survival, % (mean, SD)

Abdomen	Gentile, 2018^27^	RCS	It	2008-2018	60	41.5 ±11.3	28 ±3.0	No	AFT	No	Both	Manuel	Ce	2	187 ±35.0	MRI	12	39 ±4.4
					60	41.5 ±10.8	28 ±3.0	No	AFT	No	Both	Manuel	Ce	2	187 ±35.0	MRI	12	60.5 ±12.5
	Sforza, 2016^28^	PCS	UK	2012-2012	26	24.0 ±3.5	NR	No	AFT + implants	No	Au	Manuel	De*	1	148 ±48	2D-IS	12	72.5 ±0.8
	Small, 2014^26^	RCS	US	2009-2012	46	49 ±7.5	NR	Yes	AFT + other reconstruction	No	BR	Suction assisted	Ce	1	101, NR	3D-IS	4.6	45, NR
	Tissiani, 2016^29^	RCT	Bz	2012-2015	8	49.8 ±10.7	25.9 ±3.3	Yes	AFT + other reconstruction	No	BR	Suction assisted	Ce	NR	111.5, NR	MRI	12.9	51.4 ±18.4
					11	49.6 ±5.4	26.3 ±2.3	Yes	AFT + other reconstruction	SVF	BR	Suction assisted	Ce	NR	134.3, NR	MRI	14.5	78.8 ± 74.9
Thighs	Liu, 2024^30^	RST	Ch	2020-2020	18	28.4 ±6.5	20.3 ±2.1	No	AFT	No	Au	Manuel	Ce	1	NR	3D-IS	6	31.1 ±13.1
										BTX	Au	Manual	Ce	1	NR	3D-IS	6	40.8 ±16.6
	Pietruski, 2021^31^	RST	Po	NR	15	31.8 ±1.5	22.4, NR	No	AFT	No	Au	Suction assisted	Se	1	145, NR	MRI	6	33.4 ±28.3
										NAC	Au	Suction assisted	Se	1	145, NR	MRI	6	45.6 ±31.0
	Small, 2014^26^	RCS	US	2009-2012	27	49 ±7.5	NR	Yes	AFT + other reconstruction	No	BR	Suction assisted	Ce	1	102, NR	3D-IS	4.6	46, NR
	Wang, 2025^32^	RCT	Ch	2017-2023	10	29.6 ±9.5	20.9 ±2.2	No	AFT	No	Both	NR	Ce	3	NR	3D-IS	6	35.0 ±5.8
					10	31.7 ±8.6	19.9 ±3.2	No	AFT	BTX	Both	NR	Ce	3	NR	3D-IS	6	43.7 ±1.7

*Abbreviations:* 2D-IS = imaging software based on 2D-photographs, 3D-IS = imaging software based on 3D-photographs, AFT = autologous fat transfer, Au = augmentation mammoplasty, BR = breast reconstruction, BTX = Botulinum-toxin, Bz = Brazil, Ce = centrifugation, Ch = China, CS = retrospective case series, CTS = comparative translational study, De = device, It = Italy, MRI = magnetic resonance imaging, NAC = N-acetylcysteine, NR = not reported, PCS = prospective cohort study, Po = Poland, PRP = Platelet-rich plasma, RCS = retrospective cohort study, RCT = randomized controlled trial, RST = randomized self-controlled clinical trial, Se = sedimentation, SVF = stromal vascular fraction, UK = United Kingdom, US = United States of America.

*Device used is the PureGraft®.The figure captions are as followed:

### Study characteristics

Seven studies comprising eleven patient groups were included. Only one study compared two donor sites; Small et al.^26^ performed a retrospective study evaluating the abdomen and thighs as donor sites. Three studies used exclusively the abdomen as donor site. Gentile et al.^27^ performed a retrospective cohort study comparing two reinjection techniques. As no consensus exists regarding the optimal technique, both groups were included.^12^^,^^13^ Sforza et al.^28^ conducted a prospective cohort study without a control group. Tissiani et al.^29^ conducted a prospective controlled study comparing unenriched AFT with SVF-enriched AFT. The other three studies used exclusively the thighs as donor site. Liu et al.^30^ conducted a randomized self-controlled trial comparing unenriched AFT to botulinum-toxin (BTX)-enriched AFT. Pietruski et al.^31^ performed a randomized self-controlled trial comparing AFT using standard tumescent fluid to AFT using N-actylcysteine (NAC)-enriched tumescent fluid. Finally, Wang et al.^32^ reported a randomized controlled trial comparing unenriched AFT and BTX-enriched AFT in patients with Poland syndrome.

The studies included 291 patients, of whom 211 received AFT using the abdomen and 80 using the thighs as donor site.^26^, ^27^, ^28^, ^29^, ^30^, ^31^, ^32^ Mean age ranged from 24 ± 3.5 to 50 ±10.7 years.^26^, ^27^, ^28^, ^29^, ^30^, ^31^, ^32^ Mean BMI ranged from 20 ±2.1 kg/m^2^ to 28 ±3.0 kg/m^2^.^27^^,^^29^, ^30^, ^31^, ^32^ One study reported a normal BMI without specifying exact values.^26^ Indications for AFT varied: two studies included both breast reconstructions and augmentations,^27^^,^^32^ three studies focused exclusively on augmentations,^28^^,^^30^^,^^31^ and two studies evaluated AFT for breast reconstruction in combination with flap- or implant-based reconstruction.^26^^,^^29^ Only two studies included irradiated breasts.^26^^,^^29^

Fat was harvested by manual suction in three studies,^27^^,^^28^^,^^30^ and by suction-assisted liposuction in three studies.^26^^,^^29^^,^^31^ All studies infiltrated the donor site with tumescent fluid prior to liposuction.^26^, ^27^, ^28^, ^29^, ^30^, ^31^, ^32^ Lipoaspirate was processed exclusively by centrifugation in five studies,^26^^,^^27^^,^^29^^,^^30^^,^^32^ by sedimentation in one study,^31^ and using a commercial filtration device (PureGraft®, Bimini Health Tech, Solana Beach, California, United States of America) in one study.^28^ In all studies, fat was injected in small aliquots through tunnels in multiple planes.^26^, ^27^, ^28^, ^29^, ^30^, ^31^, ^32^ Mean total injected volume ranged from 101 to 187 mL.^26^, ^27^, ^28^, ^29^^,^^31^ Two studies did not report mean injection volumes: Liu et al.^30^ reported injecting 160-250 ml lipoaspirate and Wang et al.^32^ reported that the goal was to correct a mean volume deficit of around 168 ml in both enriched and unenriched groups. Since both studies intended an injected volume of at least 100 mL, they were included.

Measurement techniques for fat graft survival varied across studies. Three studies used magnetic resonance imaging to calculate the amount of retained fat.^27^^,^^29^^,^^31^ The other studies used imaging software; three used imaging software based on 3-dimensional pictures^26^^,^^30^^,^^32^ and *Sforza et al.*^28^ used imaging software based on 2-dimensional pictures (Crisalix®, Lausanne, Switzerland).

### Risk of bias assessment

**Fig. 2:**
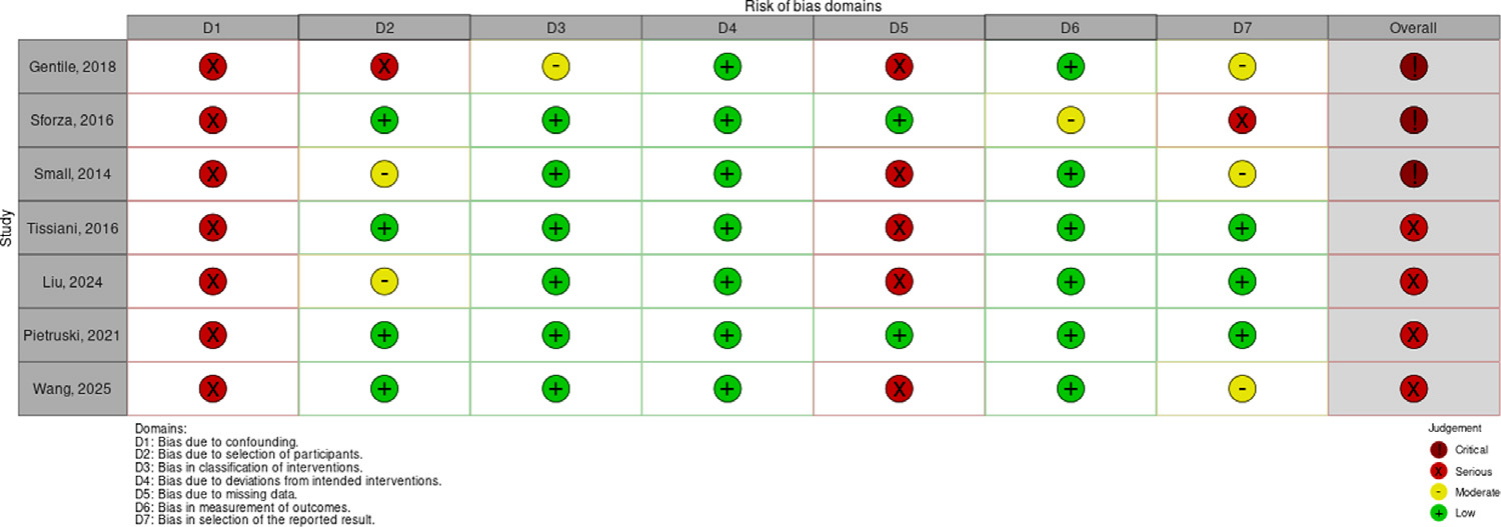
Risk of bias assessment per domain per study.

**Fig. 3:**
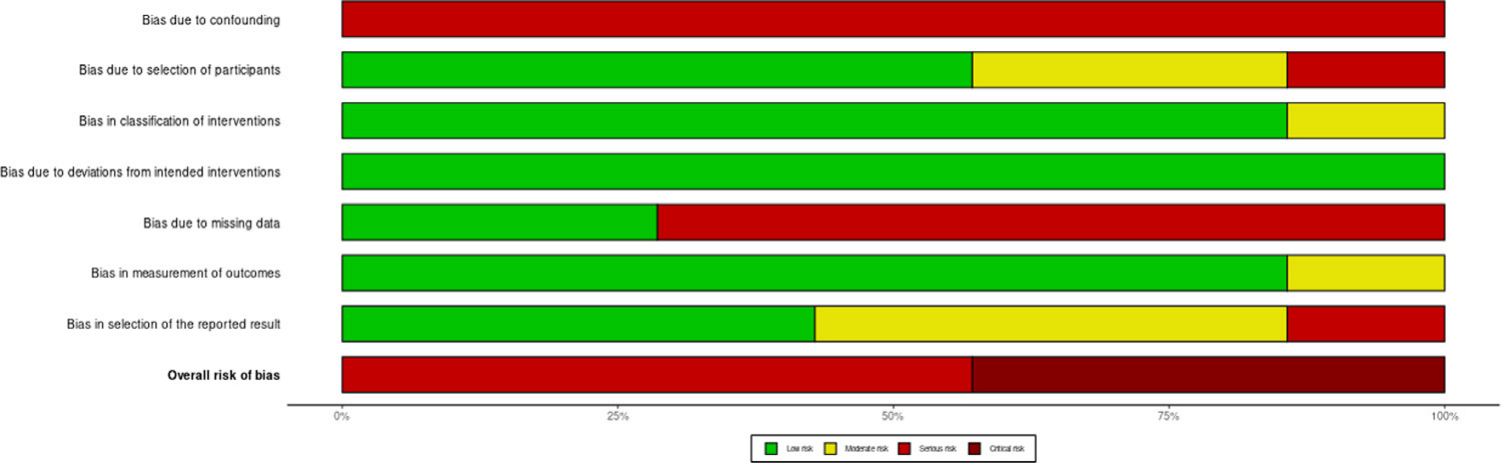
Risk of bias assessment per domain.

Risk of bias was assessed using the ROBIN-I V2 tool (Figs. 2 and 3.^33^). In their ability to answer our research question, all included studies were judged to have a serious or critical risk of bias.

All studies, even the randomized studies, were judged to have a serious risk of bias due to confounding. For the randomized trials, this was due to the reviewers’ decision to assess treatment arms separately, thereby negating the protective effect of randomization.^29^, ^30^, ^31^, ^32^ The non-randomized studies were likewise assessed as having serious risk of bias.^26^, ^27^, ^28^ The only study comparing two donor sites presented baseline characteristics (e.g. age and BMI) for the overall cohort rather than by study group.^26^ The remaining studies provided unclear rationales for treatment allocation, reported baseline data only for the total cohort, failed to analyze baseline differences, and/or included uncontrolled differences in surgical indications between groups.^27^^,^^28^

Risk of bias in selection of participants varied from low to serious. Two studies were assessed to have a moderate risk: *Liu et al.*^30^ created a possible selection bias due to postoperative BMI changes being an exclusion criterium, and *Small et al.*^26^ based donor site selection on patients preference. The article by *Gentile et al.*^27^ was judged to have a serious risk as it was unclear how their control group was selected.

Most studies were judged to have a serious risk of bias due to missing data.^26^^,^^27^^,^^29^^,^^30^^,^^32^ Most of these studies either did not report the extent of missing data or (likely) performed a complete case analysis.^26^^,^^27^^,^^32^ Two studies reported the extent of missing data but failed to report reasons for missingness, which may have depended on the true value of the outcome.^29^^,^^30^

Most studies were considered to have a low risk of bias in outcome measurement, as they used objective assessment methods and assessors were either blinded or relied on computer-generated results.^26^^,^^27^^,^^29^, ^30^, ^31^, ^32^ Only *Sforza et al*.^28^ was assessed as having a moderate risk of bias, due to the use of imaging software based on 2D-photographs, a less reliable measurement method compared to MRI or 3D-imaging software.^34^

Risk of bias due to selective reporting varied across studies. Three studies adhered to a predefined plan and were therefore assessed as having a low risk of bias.^29^, ^30^, ^31^ Studies that did not refer to a predefined plan but showed no evidence of selective reporting based on multiple analyses were assessed as a moderate risk of bias.^26^^,^^27^ In addition, *Wang et al.*^32^ did report a predefined plan, but it lacked a prespecified analysis plan and was therefore also rated as moderate risk of bias. *Sforza et al.*^28^ demonstrated indications of selective reporting found through multiple analyses and was consequently assessed as having a serious risk of bias.

### Fat graft survival rate

**Fig. 4:**
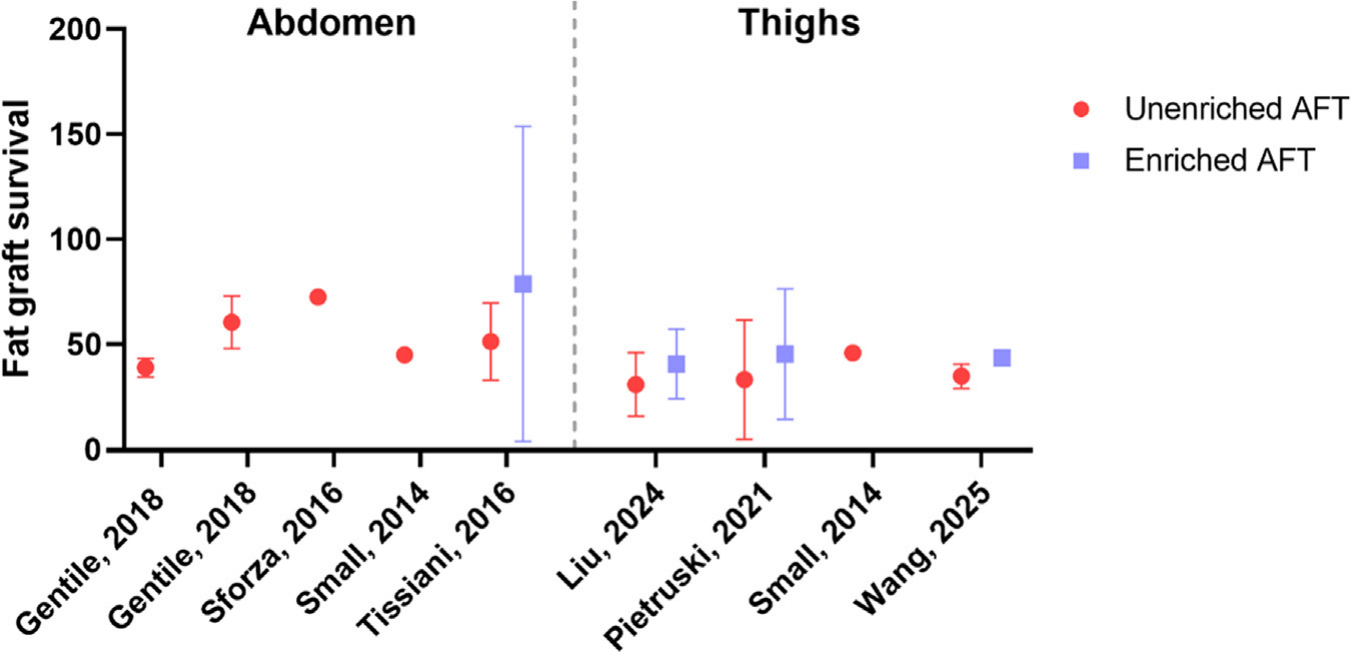
Mean fat graft survival rate (SD) per donor site per group at study end-point.

At study endpoints, reported fat graft survival rates for unenriched AFT ranged from 39% to 73% for abdominal donor sites^26^, ^27^, ^28^, ^29^ and from 31% to 46% for thigh donor sites.^26^^,^^30^, ^31^, ^32^ For enriched AFT, a fat graft survival rate of 79% was reported using abdominal donor sites,^29^ and a graft survival ranging from 41% to 46% for thigh donor sites.^30^, ^31^, ^32^ Fat graft survival rates are presented in Fig. 4. End points varied between 4.6 to 14.5 months.^26^, ^27^, ^28^, ^29^, ^30^, ^31^, ^32^ Only *Small et al*.^26^ directly compared the abdominal and thigh donor sites, reporting survival rates of 45% and 46%, respectively, at 4.6 months postoperatively, with no statistically significant difference (P > 0.05).

Meta-analyses were conducted for the studies using unenriched AFT. The study by *Small et al.**^26^* was excluded because standard deviations were not reported and the authors were unable to provide underlying data. Studies using the abdomen reported graft survival rates at 12 months postoperatively, whereas studies using the thighs reported at 6 months postoperatively. Subsequently, direct statistical comparison between the donor sites was not feasible due to time-related bias.

**Fig. 5:**
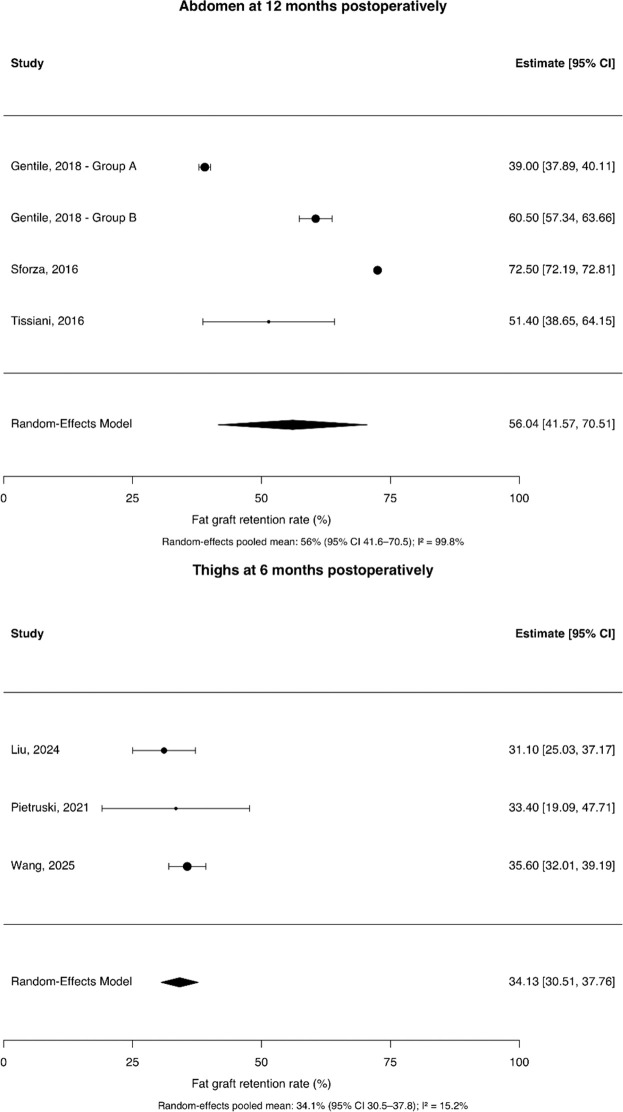
Forest plots per donor site of the fat graft survival rates at study end-point using unenriched AFT.

For abdominal donor sites, three studies comprising four groups^27^, ^28^, ^29^ were included in the meta-analysis. Substantial heterogeneity was observed (*I^2^* = 99.8%) and the pooled mean fat graft survival was estimated at 56.04% (95% CI, 41.57-70.51). For the thigh donor sites, three studies^30^, ^31^, ^32^ were included, demonstrating low heterogeneity (*I^2^* = 15.2%) with a pooled mean fat graft survival of 34.13% (95% CI, 30.51-37.76). Forest plots are presented in Fig. 5.

## Discussion

With the expanding use of AFT in breast reconstruction, interest in improving the predictability of volumetric outcomes has increased. This systematic review evaluated the effect of donor site selection on fat graft survival. Seven studies compromising 291 patients were included.^26^, ^27^, ^28^, ^29^, ^30^, ^31^, ^32^ When the abdomen was used as donor site, fat graft survival for unenriched AFT ranged from 39% to 73% for abdominal donor sites,^26^, ^27^, ^28^, ^29^ and the mean survival was estimated to be 56.04% (95% CI, 41.57-70.51, *I^2^* = 99.8%) at 12 means postoperatively, although this should be interpreted with caution given the very high I^2^ statistic. When the thighs were used as donor site, fat graft survival ranged from 31% to 46%^26^^,^^30^, ^31^, ^32^ with an estimated pooled mean of 34.13% (95% CI, 30.51-37.76) at 6 means postoperatively. For enriched AFT, fat graft survival rates were 79% using the abdomen and 41% to 46% for using the thighs as donor sites.^30^, ^31^, ^32^ Fat graft survival therefore appears slightly higher with abdominal donor sites compared with thigh donor sites for unenriched AFT, although the only study directly comparing these sites found no statistically significant difference.^26^ Given the small sample sizes in most study groups, the substantial observed heterogeneity in the abdomen group, risk of time bias and low methodological quality of most studies, these findings should be interpreted with caution and no firm conclusions can be drawn.

Adipose-derived stem cells (ADSCs) play a key role in fat graft survival.^17^^,^^19^ As ADSC concentrations vary across anatomical regions, donor site selection may influence graft survival.^20^ However, although ADSC concentrations are higher in the abdomen compared to the thighs, proliferation capacity appears to be comparable.^35^ Moreover, ADSC viability may be age-dependent: in individuals younger than 45 years higher viability is observed in the lower abdomen compared to older individuals. The same study found no difference between the age groups regarding viability in the thighs.^36^ In this systematic review, the abdomen appeared to be associated with higher graft survival. However, most included studies reported mean patient ages below 45 years, which may partly explain this observed difference.

The included studies differed in applied techniques. Three studies used manual aspiration and three used suction-assisted liposuction. Although suction-assisted liposuction has been suggested to be more traumatic to adipose tissue, *in vitro* analyses have shown no difference in cell viability.^37^ This is consistent with the findings of the present review, as studies using suction-assisted liposuction reported graft survival rates comparable to those using manual aspiration.^26^^,^^29^^,^^31^ In all studies, fat was reinjected in multiple tunnels across multiple planes within the breast. However, comparison of injection technique was limited, as the level of technical detail varied substantially between studies, with some providing extensive descriptions and others merely referring to multiplanar tunnel injection. *Gentile et al.**^27^* compared two reinjection techniques: the Coleman technique and the Gentle technique. Both techniques involved multiplanar, tunnel-based injection, and differed primarily in injection speed. Comparison of the techniques resulted in survival rates of 39% en 61%, respectively, a statistically significant difference. However, these rates are consistent with the rates reported in the other included studies.

Differences in techniques and reported survival rates could also reflect individual learning curves of performing surgeons. None of the included studies, including *Gentile et al.**^27^* addressed this factor. However, *Rijkx et al.*^38^ demonstrated that surgeons require multiple AFT procedures to learn the optimal fat reinjection volume, which is critical for graft retention. With increasing experience, surgeons tend to develop their own preferred technique, which may influence outcomes.

The role of enrichment remains debated. A survey among international plastic surgeons found that only 12.9% and 9.7% of the respondents routinely used PRP- and SVF-enriched AFT, respectively.^15^ A 2021 meta-analysis reported higher retention rates for SVF- and PRP-enriched AFT compared with unenriched AFT in breast reconstruction. However, enrichment was associated with an increased risk of complications and did not reduce the number of procedures required.^39^ In the studies included in this review, enriched AFT yielded higher graft survival than unenriched AFT, but results were in the same range.

Measurement methods for graft survival varied across studies. Volumetric measurement is influenced by the imaging modality used, patient positioning and state of respiration.^40^, ^41^, ^42^ MRI is considered the golden standard for volumetric assessment. While 3D-imaging software reports lower volumes than MRI, it provides reliable and reproducible measurements and is therefore suitable for follow-up.^43^ *Sforza et al*.^28^ used an anthropometric measurement tool based on 2-dimensional photographs. Although practical and inexpensive, this method is less accurate than MRI and 3D-imaging software and has been largely abandoned as current practice.^34^^,^^40^

In addition to technique, patient characteristics such as age, BMI, preoperative radiotherapy, preoperative breast volume and previous breast surgeries influence graft survival.^5^^,^^44^, ^45^, ^46^, ^47^ Included populations varied widely in age, with a mean age of 24 ± 3.5 to 50 ±10.7 years,^26^, ^27^, ^28^, ^29^, ^30^, ^31^, ^32^ with the lowest age reported by *Sforza et al*.,^28^ possibly contributing to their higher survival rate. BMI ranged from 20 ±2.1 kg/m^2^ to 28 ±3.0 kg/m^2^.^27^^,^^29^, ^30^, ^31^, ^32^ Although higher BMI is generally associated with reduced graft retention, this effect is not observed in patients with BMI < 30 kg/m^2^,^44^^,^^45^ as was reported in all included studies. Pre-operative radiotherapy is associated with reduced graft retention,^5^ yet the two studies including irradiated breasts did not report reduced survival rates.^26^^,^^29^ Larger preoperative breast volume has been associated with higher fat graft survival.^47^ In contrast, prior breast surgery may increase tissue fibrosis, resulting in higher interstitial fluid pressure. Too much interstitial pressure is associated with less fat graft survival.^17^ In breast augmentation, preoperative volume is typically larger and fibrosis less extensive than in post-mastectomy reconstructions. However, none of the studies focused exclusively on total breast reconstruction using AFT.

The studies showed heterogeneity in terms of study end-points, ranging from 4.6 to 14.5 months.^26^, ^27^, ^28^, ^29^, ^30^, ^31^, ^32^ Some research suggests that graft retention stabilizes at three months,^14^^,^^48^ whereas other research suggests fat stabilizes after six months.^11^ Only *Small et al.*^26^ reported a survival rate before 6 months. Consequently, their survival rates might be an overestimation.

This review is limited by the small sample size, substantial heterogeneity and generally low-quality evidence. Only one study directly comparing donor sites was identified; the remaining studies addressed different research questions. Heterogeneity was observed in patient age, applied techniques, the timing and methods of outcome measurement. Meta-analysis showed substantial heterogeneity in reported outcomes and standard deviations between the studies using the abdomen as donor site. Therefore, this pooled estimated mean should be interpreted with extreme caution.

This systematic review was conducted in accordance with the Cochrane Handbook and PRISMA guidelines. All procedures were independently performed by the first two authors with discrepancies resolved through discussion until consensus was reached. Quality assessment was verified by the third author, who has great expertise in this field. Efforts to reduce publication bias included imposing no restrictions on publication status or language.

To conclude, AFT is an increasingly recognized and promising technique for total breast reconstruction. However, its widespread adaptation is limited by the unpredictable survival of transplanted fat. Among factors proposed to influence graft survival, donor site selection has received attention. Currently, donor site selection is primarily guided by the availability of adipose tissue and patient preference. This systematic review assessed the impact of donor site selection on the fat graft survival, identifying seven studies that used fat harvested from the abdomen and/or thighs. All studies were of low methodological quality to answer this particular research questions. Even though there is a trend suggesting higher survival with abdominal donor sites, no definitive conclusions can be drawn. Based on this systematic review, no donor site can be recommended over the other based on fat graft survival. High-quality, standardized research comparing different donor sites in large-volume breast autologous fat transfer is warranted to establish evidence-based clinical guidance.

## Ethical approval

Not required.

## Funding

None.

## Registration

PROSPERO 2025 CRD420251141335. Available from https://www.crd.york.ac.uk/PROSPERO/view/CRD420251141335

## Declaration of competing interest

None declared.

## Appendix Supplementary materials

Image, application 1
