# Supplementary material for: Impact of donor site on fat graft survival in autologous fat transfer to the breast: A systematic review
Source: JPRAS Open. 2026 Jun 17;51:217–29. doi: 10.1016/j.jpra.2026.06.002 (PMC13382271; doi:10.1016/j.jpra.2026.06.002)
Supplement: Supplementary file 1 [file mmc1.docx]

Impact of Donor Site on Fat Graft Survival in Autologous Fat Transfer to the Breast: A systematic review

Kyrah S. Goeree MD^1,2^, Wessel B.W. van der Venne MD^2,3^, E. Visser PhD^4^, Andrzej Piatkowski MD, PhD^2,3^, Chantal M. Moues-Vink MD, PhD^1^

**Supplemental Table 1: Comprehensive search strategy per database**

| **Database** | **Search strategy** | **Number of results** |
| --- | --- | --- |
| MEDLINE | (exp Adipose Tissue/tr or exp Adipocytes/tr or ((exp "Autografts"/ or exp "tissue and organ harvesting"/ or exp "Transplantation, Autologous"/) and (exp "Adipose Tissue"/ or exp "Adipocytes"/ or (fat or fats or lipo* or lipid* or adipose).ti,ab,kf.)) or exp "lipectomy"/ or (((fat or fats or lipo* or lipid* or adipose) adj3 (trans* or injection* or graft* or autograft* or autotrans*)) or lipoinjection* or lipomodel* or lipotrans* or lipoaspirat* or liposuct* or lipectom* or lipofil* or AFT).ti,ab,kf.) and (exp "Mammaplasty"/ or exp Breast/su or (((Reconstruct* or Augment* or Oncoplasti*) adj4 Breast*) or mammoplast* or mastectom* or mastopex*).ti,ab,kf. or (exp "Transplant Donor Site"/ or ("donor site*" or "harvest site*").ti,ab,kf.))  NOT (comment or letter or editorial).pt | 2.370 + 189 |
| Cochrane | ((((fat or fats or lipo* or lipid* or adipose) NEAR/2 (trans* or injection* or graft* or autograft* or autotrans*)) or lipoinjection* or lipomodel* or lipotrans* or lipoaspirat* or liposuct* or lipectom* or lipofil* or AFT):ti,ab,kw) AND ((((Reconstruct* or Augment* or Oncoplasti* or surger*) NEAR/4 Breast*) or mammoplast* or mastectom* or mastopex*):ti,ab,kw  OR (donor NEXT site* or harvest NEXT site*):ti,ab,kw) | 137 + 18 |
| Embase | ((((exp "Autograft"/ or exp graft harvesting/ or exp "autotransplantation"/) and (exp "Adipose Tissue"/ or exp "Adipocyte"/ or (fat or fats or lipo* or lipid* or adipose).ti,ab,kf.)) or exp "lipectomy"/ or (((fat or fats or lipo* or lipid* or adipose) adj2 (trans* or injection* or graft* or autograft* or autotrans*)) or lipoinjection* or lipomodel* or lipotrans* or lipoaspirat* or liposuct* or lipectom* or lipofil* or AFT).ti,ab,kf.) AND (exp "Breast reconstruction"/ or breast/su or (((Reconstruct* or Augment* or Oncoplasti* or surger*) adj4 Breast*) or mammoplast* or mastectom* or mastopex*).ti,ab,kf. OR (exp donor site/ or ("donor site*" or "harvest site*").ti,ab,kf.))) NOT ("Conference paper" OR "Conference Abstract" OR "Conference Review" OR Letter OR Editorial) | 2.922 + 186 |
| Scopus | ( ( TITLE-ABS-KEY ( ( ( fat OR fats OR lipo* OR lipid* OR adipose ) W/1 ( trans* OR injection* OR graft* OR autograft* OR autotrans* ) ) OR lipoinjection* OR lipomodel* OR lipotrans* OR lipoaspirat* OR liposuct* OR lipectom* OR lipofil* OR aft ) ) AND ( TITLE-ABS-KEY ( ( ( reconstruct* OR augment* OR oncoplasti* OR surger* ) W/3 breast* ) OR mammoplast* OR mastectom* OR mastopex* ) OR TITLE-ABS-KEY ( "donor site*" OR "harvest site*" ) ) ) AND NOT DOCTYPE ( le OR ed OR cp OR cr ) | 3.052 + 634 |

**Supplemental Table 2: Data-extraction table**

|  | Study design | Study population | Intervention | Comparators | Outcomes |
| --- | --- | --- | --- | --- | --- |
| Author, publication year | Published or unpublished  Study design  Country  Centre  Start date, end date | Number of patients/breasts included  Age (mean, SD)  BMI (mean, SD)  Chest circumference (mean, SD)  Estrogen levels/hormonal substitution  Patients with diabetes mellitus present  Irradiated breasts included  Follow-up time, months (mean, SD) | Surgery type (AFT, AFT + flap, AFT + implant)  Indication (cosmetic, reconstructive)  Harvesting technique used  Processing technique used  Injection technique used  Number of sessions (mean, SD)  Volume of lipofilling per session, ml (mean, SD)  Total volume of lipofilling, ml (mean, SD) | Donor sites used | Measurement of outcome  Time point, months  Graft survival (mean %, SD) |

**Supplemental Table 3: Detailed extracted data regarding study design and study population.**

|  |  | Study design | | | Study population | | | | | | | | | |
| --- | --- | --- | --- | --- | --- | --- | --- | --- | --- | --- | --- | --- | --- | --- |
| Donor site used | First author, publication year | Study design | Country | Start year – end year | Number of included patients | Number of included breasts | Age, years (mean, SD) | BMI, kg/m^2^ (mean, SD) | Chest circumference (mean, SD) | Use of hormonal substituion | Circulation oestrogen levels | Diabetes mellitus included? | Radiotherapy included? | Follow-up, months (mean, SD) |
| Abdomen | **Gentile, 2018^1^** | Retrospective cohort study | Italy | 2008-2018 | 60 | 120 | 41.5 ±11.3 | 28 ±3.0 | NR | NR | NR | Excluded | No | 60, NR |
|  |  |  |  |  | 60 | 120 | 41.5 ±10.8 | 28 ±3.0 | NR | NR | NR | Excluded | No | 60, NR |
|  | **Sforza, 2016^2^** | Prospective cohort study | UK | 2012 - 2012 | 26 | 50 | 24.0 ±3.5 | NR | NR | NR | NR | Excluded | No | 12, NR |
|  | **Small, 2014^3^** | Retrospective cohort study | USA | 2009 - 2012 | 46 | 66 | 49 ±7.5 | NR | NR | NR | NR | NR | Yes | 5, NR |
|  | **Tissiani, 2016^4^** | Randomized controlled trial | Brazil | 2012-2015 | 8 | 8 | 49.8 ±10.7 | 25.9 ±3.3 | NR | NR | NR | NR | Yes | 16 ±7.3 |
|  |  |  |  |  | 11 | 11 | 49.6 ±5.4 | 26.3 ±2.3 | NR | NR | NR | NR | Yes | 36 ±7.2 |
| Thighs | **Liu, 2024^5^** | Randomized self-controlled trial | China | 2020 - 2020 | 18 | 18 | 28.4 ±6.5 | 20.3 ±2.1 | NR | NR | NR | NR | No | 6, NR |
|  |  |  |  |  |  | 18 |  |  |  |  |  |  |  |  |
|  | **Pietruski, 2021^6^** | Randomized self-controlled trial | Poland | NR | 15 | 15 | 31.8 ±1.5 | 22.4, NR | NR | NR | NR | NR | No | 6, NR |
|  |  |  |  |  |  | 15 |  |  |  |  |  |  |  |  |
|  | **Small, 2014^3^** | Retrospective cohort study | US | 2009 - 2012 | 27 | 43 | 49 ±7.5 | NR | NR | NR | NR | NR | Yes | 5, NR |
|  | **Wang, 2025^7^** | Randomized controlled trial | China | 2017-2023 | 10 | 10 | 29.6 ±9.5 | 20.9 ±2.2 | NR | NR | NR | NR | No | 6, NR |
|  |  |  |  |  | 10 | 10 | 31.7 ±8.6 | 19.9 ±3.2 | NR | NR | NR | NR | No | 6, NR |

Abbreviations: NR = not reported, UK = United Kingdom, USA = United States of America.

**Supplemental Table 4: Detailed extracted data regarding intervention and outcomes.**

|  |  | Intervention | | | | | | | | Outcome | | |
| --- | --- | --- | --- | --- | --- | --- | --- | --- | --- | --- | --- | --- |
| Donor site used | First author, publication year | Surgery type | Enrichment | Indication (reconstructive, augmentation) | Harvesting technique (manuel/device) | Processing technique | Number of sessions (mean) | Injection technique | Total injection volume, ml (mean, SD) | Measurement of outcome | Time of measurement, months | Fat graft survival, % (mean, SD) |
| Abdomen | **Gentile, 2018^1^** | AFT | No | Both | Manuel | Centrifugation | 2 | Multiple planes and tunnels | 187 ±35.0 | MRI | 12 | 39 ±4.4 |
|  |  | AFT | No | Both | Manuel | Centrifugation | 2 | Multiple planes and tunnels ** | 187 ±35.0 | MRI | 12 | 60.5 ±12.5 |
|  | **Sforza, 2016^2^** | AFT + implants | No | Augmentation | Manuel | Device* | 1 | Multiple planes and tunnels | 148 ±48 | 2D-IS | 12 | 72.5 ±0.8 |
|  | **Small, 2014^3^** | AFT + other reconstruction | No | Reconstructive | Suction assisted | Centrifugation | 1 | Multiple planes and tunnels | 101, NR | 3D-IS | 0.5 | 82, NR |
|  |  |  |  |  |  |  |  |  |  |  | 1.6 | 63, NR |
|  |  |  |  |  |  |  |  |  |  |  | 4.6 | 45, NR |
|  | **Tissiani, 2016^4^** | AFT + other reconstruction | No | Reconstructive | Suction assisted | Centrifugation | NR | Multiple planes and tunnels | 111.5, NR | MRI | 12.9 | 51.4 ±18.4 |
|  |  | AFT + other reconstruction | SVF | Reconstructive | Suction assisted | Centrifugation | NR | Multiple planes and tunnels | 134.3, NR | MRI | 14.5 | 78.8 ± 74.9 |
| Thighs | **Liu, 2024^5^** | AFT | No | Augmentation | Manuel | Centrifugation | 1 | Multiple planes and tunnels | NR | 3D-IS | 3 | 35.3 ±15.1 |
|  |  |  |  |  |  |  |  |  |  |  | 6 | 31.1 ±13.1 |
|  |  |  | BTX | Augmentation | Manual | Centrifugation | 1 | Multiple planes and tunnels | NR | 3D-IS | 3 | 51.7 ±19.4 |
|  |  |  |  |  |  |  |  |  |  |  | 6 | 40.8 ±16.6 |
|  | **Pietruski, 2021^6^** | AFT | No | Augmentation | Suction assisted | Sedimentation | 1 | Multiple planes and tunnels | 145, NR | MRI | 6 | 33.4 ±28.3 |
|  |  |  | NAC | Augmentation | Suction assisted | Sedimentation | 1 | Multiple planes and tunnels | 145, NR | MRI | 6 | 45.6 ±31.0 |
|  | **Small, 2014^3^** | AFT + other reconstruction | No | Reconstructive | Suction assisted | Centrifugation | 1 | Multiple planes and tunnels | 102, NR | 3D-IS | 0.5 | 86, NR |
|  |  |  |  |  |  |  |  |  |  |  | 1.6 | 63, NR |
|  |  |  |  |  |  |  |  |  |  |  | 4.6 | 46, NR |
|  | **Wang, 2025^7^** | AFT | No | Both | NR | Centrifugation | 3 | Multiple planes and tun0nels | NR | 3D-IS | 3 | 46.7 ±4.4 |
|  |  |  |  |  |  |  |  |  |  |  | 6 | 35.0 ±5.8 |
|  |  | AFT | BTX | Both | NR | Centrifugation | 3 | Multiple planes and tunnels | NR | 3D-IS | 3 | 55.0 ±4.4 |
|  |  |  |  |  |  |  |  |  |  |  | 6 | 43.7 ±1.7 |

Abbreviations: 2D-IS = imaging software based on 2D-photographs, 3D-IS = imaging software based on 3D-photographs, AFT = autologous fat transfer, BTX = Botulinum-toxin, MRI = magnetic resonance imaging, NAC = N-acetylcysteine, NR = not reported, PRP = Platelet-rich plasma, SVF = stromal vascular fraction.

* Device used is the PureGraft®.

** In the article, they describe their own technique, called the “Gentle technique”. This technique focusses on slow re-injection of the fat using controlled movements. However, the fat is still re-injected in multiple planes using multiple tunnels.

**Supplemental Table 5: PRISMA 2020 Checklist^8^**

| **Section and Topic** | **Item #** | **Checklist item** | **Location where item is reported** |
| --- | --- | --- | --- |
| **TITLE** | | |  |
| Title | 1 | Identify the report as a systematic review. | Page 1 |
| **ABSTRACT** | | |  |
| Abstract | 2 | See the PRISMA 2020 for Abstracts checklist. | Page 2 |
| **INTRODUCTION** | | |  |
| Rationale | 3 | Describe the rationale for the review in the context of existing knowledge. | Page 3 |
| Objectives | 4 | Provide an explicit statement of the objective(s) or question(s) the review addresses. | Page 3-4 |
| **METHODS** | | |  |
| Eligibility criteria | 5 | Specify the inclusion and exclusion criteria for the review and how studies were grouped for the syntheses. | Page 4-5 |
| Information sources | 6 | Specify all databases, registers, websites, organisations, reference lists and other sources searched or consulted to identify studies. Specify the date when each source was last searched or consulted. | Page 4 |
| Search strategy | 7 | Present the full search strategies for all databases, registers and websites, including any filters and limits used. | Page 4 + Supplemental Table 1 |
| Selection process | 8 | Specify the methods used to decide whether a study met the inclusion criteria of the review, including how many reviewers screened each record and each report retrieved, whether they worked independently, and if applicable, details of automation tools used in the process. | Page 4-5 |
| Data collection process | 9 | Specify the methods used to collect data from reports, including how many reviewers collected data from each report, whether they worked independently, any processes for obtaining or confirming data from study investigators, and if applicable, details of automation tools used in the process. | Page 5 |
| Data items | 10a | List and define all outcomes for which data were sought. Specify whether all results that were compatible with each outcome domain in each study were sought (e.g. for all measures, time points, analyses), and if not, the methods used to decide which results to collect. | Page 5 Supplemental Table 2 |
|  | 10b | List and define all other variables for which data were sought (e.g. participant and intervention characteristics, funding sources). Describe any assumptions made about any missing or unclear information. | Supplemental Table 2 |
| Study risk of bias assessment | 11 | Specify the methods used to assess risk of bias in the included studies, including details of the tool(s) used, how many reviewers assessed each study and whether they worked independently, and if applicable, details of automation tools used in the process. | Page 5 |
| Effect measures | 12 | Specify for each outcome the effect measure(s) (e.g. risk ratio, mean difference) used in the synthesis or presentation of results. | Page 6 |
| Synthesis methods | 13a | Describe the processes used to decide which studies were eligible for each synthesis (e.g. tabulating the study intervention characteristics and comparing against the planned groups for each synthesis (item #5)). | Page 5-6 |
|  | 13b | Describe any methods required to prepare the data for presentation or synthesis, such as handling of missing summary statistics, or data conversions. | Page 6 |
|  | 13c | Describe any methods used to tabulate or visually display results of individual studies and syntheses. | Page 6 |
|  | 13d | Describe any methods used to synthesize results and provide a rationale for the choice(s). If meta-analysis was performed, describe the model(s), method(s) to identify the presence and extent of statistical heterogeneity, and software package(s) used. | Page 6 |
|  | 13e | Describe any methods used to explore possible causes of heterogeneity among study results (e.g. subgroup analysis, meta-regression). | Page 6 |
|  | 13f | Describe any sensitivity analyses conducted to assess robustness of the synthesized results. | Page 6 |
| Reporting bias assessment | 14 | Describe any methods used to assess risk of bias due to missing results in a synthesis (arising from reporting biases). | Page 5-6 |
| Certainty assessment | 15 | Describe any methods used to assess certainty (or confidence) in the body of evidence for an outcome. | Page 6 |
| **RESULTS** | | |  |
| Study selection | 16a | Describe the results of the search and selection process, from the number of records identified in the search to the number of studies included in the review, ideally using a flow diagram. | Page 6 |
|  | 16b | Cite studies that might appear to meet the inclusion criteria, but which were excluded, and explain why they were excluded. | Page 6 |
| Study characteristics | 17 | Cite each included study and present its characteristics. | Page 7-8, Table 3. Supplemental Table 3-4 |
| Risk of bias in studies | 18 | Present assessments of risk of bias for each included study. | Page 9-10 |
| Results of individual studies | 19 | For all outcomes, present, for each study: (a) summary statistics for each group (where appropriate) and (b) an effect estimate and its precision (e.g. confidence/credible interval), ideally using structured tables or plots. | Page 10-12 |
| Results of syntheses | 20a | For each synthesis, briefly summarise the characteristics and risk of bias among contributing studies. | Page 10-12 |
|  | 20b | Present results of all statistical syntheses conducted. If meta-analysis was done, present for each the summary estimate and its precision (e.g. confidence/credible interval) and measures of statistical heterogeneity. If comparing groups, describe the direction of the effect. | Page 10-12 |
|  | 20c | Present results of all investigations of possible causes of heterogeneity among study results. | Page 10-12 |
|  | 20d | Present results of all sensitivity analyses conducted to assess the robustness of the synthesized results. | Page 10-12 |
| Reporting biases | 21 | Present assessments of risk of bias due to missing results (arising from reporting biases) for each synthesis assessed. | Page 10-12 |
| Certainty of evidence | 22 | Present assessments of certainty (or confidence) in the body of evidence for each outcome assessed. | Page 10-12 |
| **DISCUSSION** | | |  |
| Discussion | 23a | Provide a general interpretation of the results in the context of other evidence. | Page 13—14 |
|  | 23b | Discuss any limitations of the evidence included in the review. | Page 14-15 |
|  | 23c | Discuss any limitations of the review processes used. | Page 14-15 |
|  | 23d | Discuss implications of the results for practice, policy, and future research. | Page 15 |
| **OTHER INFORMATION** | | |  |
| Registration and protocol | 24a | Provide registration information for the review, including register name and registration number, or state that the review was not registered. | Page 2 |
|  | 24b | Indicate where the review protocol can be accessed, or state that a protocol was not prepared. | Page 2 |
|  | 24c | Describe and explain any amendments to information provided at registration or in the protocol. | Page 2 |
| Support | 25 | Describe sources of financial or non-financial support for the review, and the role of the funders or sponsors in the review. | Page 2 |
| Competing interests | 26 | Declare any competing interests of review authors. | Page 15 |
| Availability of data, code and other materials | 27 | Report which of the following are publicly available and where they can be found: template data collection forms; data extracted from included studies; data used for all analyses; analytic code; any other materials used in the review. | Supplementary Data |

*From:*  Page MJ, McKenzie JE, Bossuyt PM, Boutron I, Hoffmann TC, Mulrow CD, et al. The PRISMA 2020 statement: an updated guideline for reporting systematic reviews. BMJ 2021;372:n71. doi: 10.1136/bmj.n71. This work is licensed under CC BY 4.0. To view a copy of this license, visit <https://creativecommons.org/licenses/by/4.0/>

# References

1. Gentile P, De Angelis B, Di Pietro V, et al. Gentle is better: The original 'gentle technique' for fat placement in breast lipofilling. Article. *J Cutan Aesthet Surg*. 2018;11(3):120–126. doi:10.4103/JCAS.JCAS_24_18

2. Sforza M, Andjelkov K, Zaccheddu R, Husein R, Atkinson C. A preliminary assessment of the predictability of fat grafting to correct silicone breast implant-related complications. Article. *Aesthet Surg J*. 2016;36(8):886–894. doi:10.1093/asj/sjw060

3. Small K, Choi M, Petruolo O, Lee C, Karp N. Is there an ideal donor site of fat for secondary breast reconstruction? *Aesthet Surg J*. 2014;34(4):545–550. doi:<https://dx.doi.org/10.1177/1090820X14526751>

4. Tissiani LAL, Alonso N. A prospective and controlled clinical trial on stromal vascular fraction enriched fat grafts in secondary breast reconstruction. Article. *Stem Cells Intl*. 2016;20162636454. doi:10.1155/2016/2636454

5. Liu T, Qiang S, Wang N, et al. Improving the Retention Rate of Fat Grafting by Botulinum Toxin A: A Randomized, Self-controlled, Clinical Trial. Article. *Aesthet Plast Surg*. 2024;doi:10.1007/s00266-024-04342-1

6. Pietruski P, Paskal W, Paluch Ł, et al. The Impact of N-Acetylcysteine on Autologous Fat Graft: First-in-Human Pilot Study. Article. *Aesthet Plast Surg*. 2021;45(5):2397–2405. doi:10.1007/s00266-020-01633-1

7. Wang N, Wei S, Qiang S, Wang J, Zeng X, Z. Z. Autologous Fat Graft Combined With Botulinum Toxin Injection for Breast Augmentation in Poland Syndrome: A Prospective and Comparative Study. *J Cosmet Dermatol*. 2025;24(2):e70070. doi:doi:10.1111/jocd.70070

8. Page MJ, McKenzie JE, Bossuyt PM, et al. The PRISMA 2020 statement: an updated guideline for reporting systematic reviews. *BMJ*. 2021;372(n71)doi:10.1136/bmj.n71
